# Genome sequence of *Streptomyces* sp. DSM 42143

**DOI:** 10.1128/mra.00630-25

**Published:** 2025-11-04

**Authors:** Charlotte Holding, Imen Nouioui, Markus Göker, Alex Spunde, Natalia Ivanova, Rekha Seshadri, Craig Stephens

**Affiliations:** 1 Department of Biology, Santa Clara University 166590, Santa Clara, California, USA; 2 Leibniz Institute DSMZ, German Collection of Microorganisms and Cell Cultures, Braunschweig, Germany; 3 DOE Joint Genome Institute, Lawrence Berkeley National Laboratory 1666, Berkeley, California, USA; University of Strathclyde, Glasgow, United Kingdom

**Keywords:** *Streptomyces*, genome analysis, antibiotic production

## Abstract

We report the 7.9 Mb genome sequence of *Streptomyces* sp. DSM 42143, which contains a 7,807,748 bp linear chromosome and a 108,164 bp circular plasmid. AntiSMASH predicted 39 diverse biosynthetic gene clusters, suggesting that this strain may be a rich resource for antibiotic discovery.

## ANNOUNCEMENT

*Streptomyces* species (*Bacteria*, *Actinomycetota*, *Actinomycetes*, *Kitasatosporales*, *Streptomycetaceae*) are soil-dwelling Gram-positive filamentous bacteria recognized for synthesizing diverse secondary metabolites (1, 2). *Streptomyces* sp. DSM 42143 was isolated from Saudi Arabian soil in 2011 and maintained by the Leibniz Institute DSMZ culture collection. This strain was sequenced as part of the DOE Joint Genome Institute (JGI) “*Sequencing the genomes of 1000 actinobacteria strains*” project, given its potential to produce new antibiotics and other bioactive compounds (3).

*Streptomyces* sp. DSM 42143 cultures were revived from freeze-dried cells and grown at the DSMZ in liquid medium DSMZ 987 (4), incubated for 5 days at 28°C with shaking at 120 rpm. Genomic DNA was extracted at the DSMZ using the MasterPure Gram Positive DNA Purification Kit (Biosearch Technologies). The genomic DNA provided to JGI was sheared using the Megaruptor 3 (Diagenode) to generate an average fragment size of 10 kb. A library was constructed using the SMRTBell Express Template Prep Kit (Pacific Biosciences) and sequenced at JGI using Version 2.0 chemistry on the Pacific Biosciences Sequel II platform (5) using 8M v1 SMRT cells and 1 × 900 sequencing movie run times. Reads generated by the instrument (381,065 in total) were additionally filtered for collapsed smartbells (missing adapters) using icecreamfinder.sh, a script in the BB Tools suite. In total, 233,306 filtered subreads (*N*_50_ = 4988 bp) and 19,595 Circular Consensus Sequence (CCS) reads (*N*_50_ = 6.22 kb) were used as input for *de novo* assembly using Flye (v2.8.1) (6), with the parameters “–asm-coverage 100 –plasmid –genome-size 5000000t 64 –pacbio-raw,” yielding two contigs: a 7,807,748 bp linear chromosome and a 108,164 bp circular plasmid. The stem-loop rich telomere regions of linear *Streptomycete* chromosomes have been reported to be difficult to recover by genome sequencing (7). None of the six known classes of *Streptomycete* stem-loop-rich telomere sequences were identified by BLAST (8) near the putative chromosome termini of the DSM 42143 sequence, but inward-facing copies of the conserved telomeric *ttrA* gene are present (7). Circularity of the plasmid assembly was reported by Flye and confirmed with Circlator (v1.0) (9). The final assembly spans 7,915,912 bp with 122.3 × read coverage and 72.04% GC content. CheckM2 (v1.0.2) confirmed 100% completeness and 0% contamination (10).

The genome was annotated by the IMG annotation pipeline (version 5.0.23) (11) to predict a total of 7178 genes, including 7,030 protein coding genes, and 6 complete rRNA operons and 74 tRNA genes.

The DSM 42143 genome is most similar to the *Streptomyces mutabilis* JCM 4400 draft genome (GCF_014649815.1) (Average Nucleotide Identity (ANI) = 96.9%, Alignment fraction (AF) = 75.2%) (11). AntiSMASH (v.7.1.0) predicted 39 biosynthetic gene clusters (BGCs) (Table 1), including polyketide synthases, non-ribosomal peptide synthetases, and ribosomally synthesized post-translationally-modified peptide (RiPP) gene clusters often associated with antibiotic production (12). A PKS pathway most similar to that of the fluostatin family (13) was located on the 108 kb plasmid. Other BGCs whose products were not able to be predicted may also offer opportunities for discovery of novel structures and functions.

**TABLE 1:** Predicted biosynthetic gene clusters of *Streptomyces* sp. DSM 42143

Cluster name	Total amount	Products of most similar known cluster(s)
Non-ribosomal peptide synthetases	4	Coelichelin; coelibactin; candicidin; antimycin A1B
Polyketide synthases	8	Candicidin; antimycin A1B
RiPP	7	SapB; planosporicin; citrulassin E
Terpene	7	Hopene; geosmin; albaflavenone
Alkaloid	2	Unknown
Saccharide	1	Unknown
Other	6	Desferrioxamin B/desferrioxamine E; ectoine5-dimethylallylindole-3-acetonitrile
Hybrid	3	Unknown
Unknown	1	Unknown

## Data Availability

The annotated *Streptomyces* sp. DSM 42143 sequence is freely available for analysis on the JGI Integrated Microbial Genomes and Microbiomes portal under IMG Genome ID 2926792514. This Whole Genome Shotgun project has also been deposited at NCBI under accession JAUSVD000000000. Note that the IMG and NCBI annotations can be slightly different, as they use different annotation pipelines. The raw sequencing data are available under SRA accession SRX20650023.
